# Influence of Process Parameters on Selected Properties of Ti6Al4V Manufacturing via L-PBF Process

**DOI:** 10.3390/ma17174384

**Published:** 2024-09-05

**Authors:** Janusz Kluczyński, Bartłomiej Sarzyński, Tomáš Dražan, Jakub Łuszczek, Robert Kosturek, Ireneusz Szachogłuchowicz

**Affiliations:** 1 Institute of Robots & Machine Design, Faculty of Mechanical Engineering, Military University of Technology, Gen. S. Kaliskiego St., 00-908 Warsaw, Poland; bartlomiej.sarzynski@wat.edu.pl (B.S.); jakub.luszczek@wat.edu.pl (J.Ł.); robert.kosturek@wat.edu.pl (R.K.); ireneusz.szachogluchowicz@wat.edu.pl (I.S.); 2 Department of Mechanical Engineering, Faculty of Military Technology, University of Defence, 662 10 Brno, Czech Republic; tomas.drazan@unob.cz

**Keywords:** additive manufacturing, PBF-LB/M, porosity, microhardness, Ti6Al4V, titanium, aluminum, vanadium, metal alloys, microstructure

## Abstract

This study investigates the microstructural effects of process parameters on Ti6Al4V alloy produced via powder bed fusion (PBF) using laser beam melting (LB/M) technology. The research focuses on how variations in laser power, exposure velocity, and hatching distance influence the final material’s porosity, microhardness, and microstructure. To better understand the relationships between process parameters, energy density, and porosity, a simple mathematical model was developed. The microstructure of the alloy was analyzed in the YZ plane using a confocal microscope. The study identified optimal parameters—302.5 W laser power, 990 mm/s exposure velocity, and 0.14 mm hatching distance—yielding the lowest porosity index of 0.005%. The material’s average hardness was measured at 434 ± 18 HV0.5. These findings offer valuable insights for optimizing printing parameters to produce high-quality Ti6Al4V components using PBF-LB/M technology, shedding light on the critical relationship between process parameters and the resulting microstructure.

## 1. Introduction

Additive technologies are undoubtedly a huge trend and have the potential to revolutionize manufacturing in many industries. They are particularly attractive for opening up new production possibilities supporting almost any geometry, even with the possibility of reducing many part assemblies, low material consumption and the absence of the need for a wide range of semifinished products, and last but not least the cost effectiveness of complex components [1,2,3,4,5,6,7]. There are already a large number of companies on the market producing a constant economic turnover, and the application of 3D printed parts is becoming a reality or even a necessity not only for complex technical applications but also in advanced healthcare. Thanks to the availability of advanced materials, precision, quality, shape, and structural possibilities, we can nowadays see additive technology products in the form of implants, tools, components for automotive, aerospace, or components for highly loaded military technology [8,9,10,11,12,13]. However, additive technologies have also carved out a place for themselves by providing logistical support in today’s armed conflicts. Powder bed fusion-laser beam melting (PBF-LB/M) technology can be counted among the almost conventional additive technologies for metal processing [14,15]. This technology utilizes a high-power laser to accurately melt a metal powder layer. With each step, a fresh layer of powder is deposited and melted, gradually forming the material layer by layer into the finished product [14,15]. PBF-LBM technology offers several advantages over traditional manufacturing methods, such as the capability to create complex and customized geometries with high accuracy and precision, reduced material waste, and shorter lead times. It is particularly effective for small-batch production and is cost-efficient for parts with intricate designs. However, to achieve optimal results—including the best surface roughness, mechanical properties, and microstructure—it is essential to carefully establish process parameters such as laser power, exposure velocity, hatching distance, layer height, and scanning strategy. There are a lot of research works that are focused on the development of optimal process parameters for every material available in the market that is dedicated to PBF-LB/M technology. One of the examples is a study conducted by D. Lu et al. [16], who used the following parameters in their work: laser power of 160 W, exposure velocity of 1000 mm/s, hatch spacing of 0.08 mm [7], but at the same time, those parameters were valid for an exact geometry of the parts and for an exact machine setup. In addition, it is impossible to create or predict adequate universal settings for all process parameters, as the parameters are intricately interrelated, and changing one affects many aspects of the print and its resulting form and characteristics. It must be considered that each material requires different melting conditions, depending not only on the type of material but also on its production method, respectively, the particle sizes and properties of the metal in question. As with most additive technologies, this faces problems such as residual stresses, anisotropy of mechanical properties, cracks, and porosity [14,15,16].

Titanium alloys are highly sought after in the aerospace and medical industries for their exceptional mechanical strength, corrosion resistance, and biocompatibility. Among these alloys, Ti6Al4V stands out as the most widely used due to its impressive strength-to-weight ratio, high thermal stability, low modulus of elasticity, and excellent resistance to corrosion [16,17,18,19]. Thus, titanium alloys are used not only for production but also for coating [16]. However, manufacturing Ti6Al4V components using traditional methods such as casting, forging, and machining presents significant challenges. These challenges stem from the high cost and difficulty of producing complex and customized geometries. The production process is particularly demanding due to several factors: the high melting temperature, low thermal conductivity, high strength, and resistance to plastic deformation even at elevated temperatures. Additionally, titanium exhibits chemical reactivity when heated above 500 °C, a risk of autoignition, and a low modulus of elasticity that can lead to vibrations and tooling wear. These factors collectively contribute to increased costs in the conventional production of titanium alloy products [20,21]. Therefore, the use of additive manufacturing is suitable for titanium alloy production, which can overcome these difficulties [22]. However, in some instances, post-processing, such as machining, is required for printed parts. In these cases, titanium alloys produced with PBF-LB/M technology are often found to be easier to machine compared to those manufactured using conventional methods [23,24]. It is the investigation of the microstructure of Ti6Al4V parts produced using PBF-LB/M that is critical to determining the mechanical properties and performance of the final product. Several studies have looked at the effect of sample orientation and its properties on printed samples [16,24]. Due to the medical application, intensive research and optimization of printable parameters is also underway to improve roughness, texture, and surface texture [25,26], or improve the surface quality by post-processing 3D printed titanium alloy [27]. Similarly, there was a need to investigate the post-processing of PBF-LB/M processed titanium alloys and their effect on microstructural properties [28]. Other research looks at residual stress and temperature transmittance [26]. Dareh Bagdhi et al. [24] were able to achieve porosity below 0.15% and low surface roughness Ra < 10 μm by adjusting the printing parameters at laser power 180–270 W and energy density 50–100 J/mm^3^. The hardness of the material was also found to depend on the laser power in combination with the scanning rate, as well as being able to increase the corrosion resistance by increasing the laser power and scanning rate. Rahulan et al. [1] then conducted a review of the available research, where from the list of eight different studies, the process parameters were always different in some parameters than our chosen process parameters. The review and our research have identified that researchers are exploring both lower and higher values for laser power, speed, and hatching distance compared to those considered in our study. The paper also provides a summary of the mechanical properties of Ti6Al4V titanium alloy samples produced by various technologies, demonstrating that PBF-LB/M achieves superior mechanical properties compared to conventional methods.

In the current state of the art, there is limited research on the microstructural evaluation within the given range of process parameters for PBF-LB/M technology and their integration into mathematical models. Therefore, understanding the interplay between energy density and the dependent printing parameters, as well as their impact on the porosity and microstructure of printed components, is crucial for designing and fabricating components with optimal material and mechanical properties.

## 2. Materials and Methods

Ti6Al4V material from Nikon SLM Solutions company (Nikon SLM Solutions AG, Lübeck, Germany) with a powder particles size range of 15–45 μm was used for sample fabrication. Its chemical composition (from the SLM Solution material data sheet) is presented in Table 1. This alloy is characterized by high aluminum and vanadium content, with trace amounts of iron. The other elements present in the alloy do not significantly affect its properties. Its density is 4.43 g/cm^3^.

Before starting the process, the equipment was meticulously cleaned to eliminate any residues from previous operations and materials. The Ti6Al4V material was dried in a SUSLAB-BIO-005 laboratory dryer (Adverti, Łódź, Poland) at 90 °C for 24 h prior to loading and calibration. The Ti6Al4V powder, which was gas-atomized and provided by Nikon SLM Solutions AG (Nikon SLM Solutions AG, Lübeck, Germany), served as the base material. The quality of this metal powder was assessed using a Jeol JSM-6610 scanning electron microscope (SEM) (Jeol, Tokyo, Japan).

**Figure 1:**
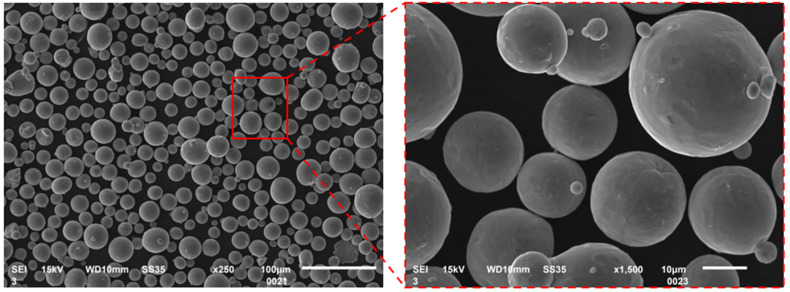
SEM images of Ti6Al4V powder.

The cube-shaped samples, each measuring 10 mm on each side, were designed using Magics 19 software (Materialise, Leuven, Belgium, version 19). This software was also used to configure the printing parameters, including laser power, layer thickness, exposure speed, and hatching distance (the spacing between laser exposure lines). Identification numbers were marked on the top surfaces of the cubes to ensure easy differentiation and avoid mix-ups. The cubes were strategically placed on the print bed to prevent interference during the printing process. The samples were manufactured using an SLM 125HL printer from Nikon SLM Solutions Group AG (Nikon SLM Solutions AG, Lübeck, Germany), as depicted in Figure 1. This printer features a build volume of 125 mm × 125 mm × 125 mm and is equipped with a laser capable of up to 400 W of power.

To appropriately select the parameters for the L-PBF process, three values were varied: laser power, exposure velocity, and hatching distance. The layer thickness during printing was kept constant at 0.03 mm. Using these parameters, the energy density per unit volume of the material was calculated using Equation (1) [29]. Calculated values were presented in Table 2.

(1) $$E_{V}=\frac{P_{L}}{h\cdot v_{s}\cdot H}$$

E_V_—energy per unit volume (J/mm^3^);

P_L_—laser power (W);

h—hatching distance (mm);

v_s_—exposure velocity (mm/s);

H—thickness of the powder layer (mm).

To protect the material from the heat generated during the sample cutting process with a conventional grinder, wire electric discharge cutting technology was utilized, specifically the Accutex AL 400SA device (AccuteX Technologies Co., Ltd., Taichung City, Taiwan). For a thorough examination, the samples were cut in two planes. Subsequently, the samples were embedded in epoxy resin and ground and polished using water paper ranging from P200 to P1000 to achieve a smooth surface finish. These grinding and polishing operations were carried out on a Struers Tegramin 30 automatic machine (Struers, Copenhagen, Denmark). Porosity measurements were performed on all 27 polished samples using a Keyence VHX-7000 digital microscope (Keyence, Osaka, Japan). The analysis of the structural quality of samples produced with varying printing parameters was conducted in two stages. First, sequentially exposed areas were stitched together, and then the ratio of the porous area detected by the optical device to the total sample area was determined. All specimens were assessed using consistent defect detection settings. The porosity value reported in this manuscript is based on a single measurement for each plane. Mounted and polished samples were etched using Keller’s reagent, which is commonly used for aluminum alloys. This reagent is a mixture of nitric acid (HNO_3_), hydrochloric acid (HCl), and hydrofluoric acid (HF). Etching times varied significantly depending on the printing parameters, requiring multiple sample preparations. Finally, microhardness tests were conducted using a Struers DuraScan device (Struers, Copenhagen, Denmark) employing the Vickers method.

According to standards and material properties, hardness measurements were conducted under a 50 g load and using a 40× objective lens magnification. The measurements were conducted on samples embedded in epoxy resin and after the grinding and polishing process. Measurements were taken 4 mm from the sample edge and then at 1 mm intervals between each subsequent measurement. The load application time was 10 s. At least six measurements were taken for each material. Before each measurement, the indenter position was adjusted to avoid structural defects, such as pores formed during printing.

Given the time-consuming nature of the parameter selection process, a Design of Experiment (DoE) analysis was employed. DoE mathematical models, renowned for their predictive capabilities regarding dependent variables, can potentially streamline the parameter selection process by reducing the number of parameter groups that need to be tested. The chosen model is the quadratic surface regression model. This model combines polynomial regression with fractional models, taking into account both the effects of independent variables on the predictor and the interactions among those variables. The independent variables include components of energy density from Formula (1), with the exception of layer thickness, which was kept constant. The dependent variable, or predictor, in this instance is the porosity value. The general form of the model is given by the following Formula (2) [30].

(2) $$y=\beta_{0}+\beta_{1}x_{1}+\beta_{2}x_{2}+\beta_{3}x_{3}{+\beta }_{11}x_{1}^{2}{+\beta }_{22}x_{2}^{2}{+\beta }_{33}x_{3}^{2}{+\beta }_{12}x_{1}x_{2}{+\beta }_{13}x_{1}x_{3}{+\beta }_{23}x_{2}x_{3}+\in$$

In Equation (2), the individual components represent the following:

y—porosity (0 ÷ 1)

x_1_—laser power [W];

x_2_—exposure velocity [mm/s];

x_3_—hatching distance [mm];

β_m_ and β_mn_ (for m = 1, 2, 3; n = 1, 2, 3)—the regression coefficients for individual variables and their product combinations;

∈—modeling residual error.

The model’s source data included actual porosity measurements from samples produced with the parameter groups specified in Table 2. Regression coefficients were computed using the least squares method. The second phase involved creating additional combinations based on a fractional factorial design 3^3^ (where each of the three independent variables was set to three different values) and incorporating these results into the model. Additionally, R^2^ and *p*-values were calculated. The R^2^ coefficient indicates how well the statistical model and its predictors explain the variability of the target quantity. The *p*-value, derived from ANOVA analysis, assesses the statistical significance of the terms, with significance considered at *p* < 0.05. All calculations and visualizations were performed using Statistica software (TIBCO Software Inc., Palo Alto, CA, USA, version 13.3). The goal of this model is to identify the parameter set that achieves a porosity value close to 0% for the model elements. The results of the statistical model provide guidance for selecting parameter groups to be tested in future stages of research on the material. Modeling residual error (∈) (refers to the difference between the observed values and the values predicted by a model. It represents the error that remains after the model has been fitted to the data. In other words, it measures how well the model predicts the actual data. A smaller residual error indicates a better fit of the model to the data, while a larger residual error suggests that the model does not adequately capture the relationship between the variables.

## 3. Results and Discussion

### 3.1. Powder Analysis

The particle analysis of the powder utilized for sample printing was conducted utilizing a Tescan Mira4 scanning electron microscope (Tescan, Brno, Czech Republic). A scale bar is included in the lower right corner. From this scale and the image, the powder particle size range, as specified by the manufacturer, can be observed. The powder particles exhibit a spherical, regular shape. This characteristic of the particles positively impacts the additive manufacturing process by facilitating the laser melting of successive powder layers. The regularity and sphericity of the particles aid in evenly spreading a uniform layer of consistent thickness. Additionally, the spherical nature of the powder does not adversely affect the recoater components, such as the “squeegee” made of soft rubber.

### 3.2. Porosity Measurement

**Figure 2:**
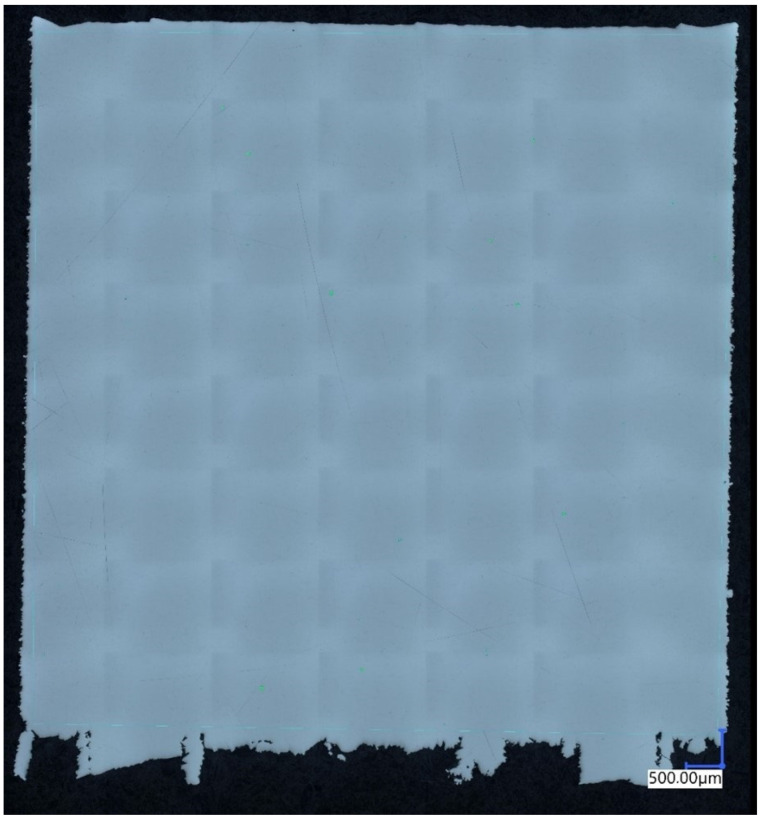
Porosity of the material manufactured using the 7th parameter group.

**Figure 3:**
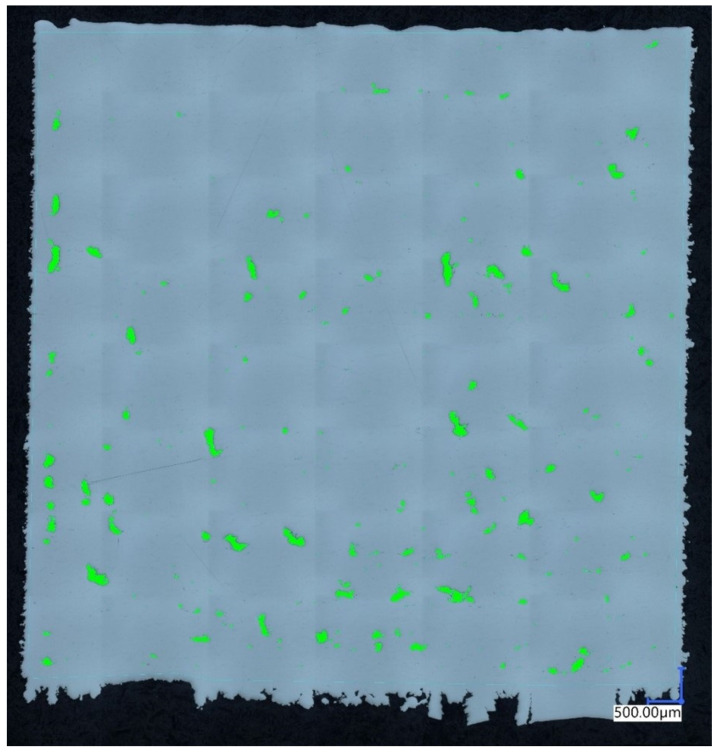
Porosity of the material manufactured using the 11th parameter group.

During the analysis of the impact of printing parameters on the material structure quality and consequently its mechanical properties, porosity plays a crucial role. Therefore, it is the primary property examined in any study. In the tested samples, porosity values ranged from 1.54% to 0.005%. The largest average pore size among all parameters was 1.27 mm, while the smallest was 0.11 mm. The lowest porosity was achieved by a sample produced using the set of parameters labeled as series 7. The highest porosity value of 1.54% was obtained by a sample produced using the parameters from series 11. The specific parameters for these series are presented in Table 3. The material porosity can be observed in Figure 2 and Figure 3.

Additionally, the uniformity of pore distribution is of paramount importance when analyzing the impact of printing parameters on material structure quality. In samples with lower porosity, the structure was more homogeneous, which contributed to better mechanical properties of the material. Conversely, higher porosity often led to the formation of undesirable defects, potentially reducing the strength and durability of the final products. The analysis of Figure 2 and Figure 3 allows for a detailed examination of pore morphology, which is essential for further optimization of the printing parameters.

Analyzing Figure 2, one can observe a high-quality, homogeneous material structure. It is difficult to identify porous areas, as confirmed by measurements conducted using digital microscope software. A scale indicating a value of 500 μm in both the vertical and horizontal directions is located in the bottom right corner. The isolated fragments of material at the bottom of the sample are remnants of supports used to facilitate the separation of the sample from the working platform of the SLM125 HL machine (Nikon SLM Solutions AG, Lübeck, Germany.

**Figure 4:**
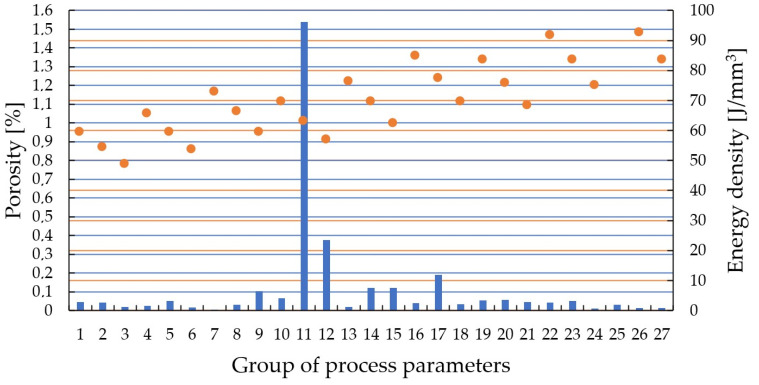
Porosity (**left**) and energy density (**right**) of all samples.

In contrast, analyzing Figure 3, a significant amount of green color representing porous areas is easily noticeable. The pores typically exhibit a non-homogeneous, slightly elongated structure. Such structural defects are caused by the lack of fusion of individual powder grains, known as the “keyhole” effect. In this case, pores resulting from trapped gas particles (gas porosity) are in the minority. A scale measuring 500 μm is also placed in the bottom right corner. Porosity (blue columns) and energy density (orange dots) of all samples were presented in the chart in Figure 4.

### 3.3. Microstructural Analysis

**Figure 5:**
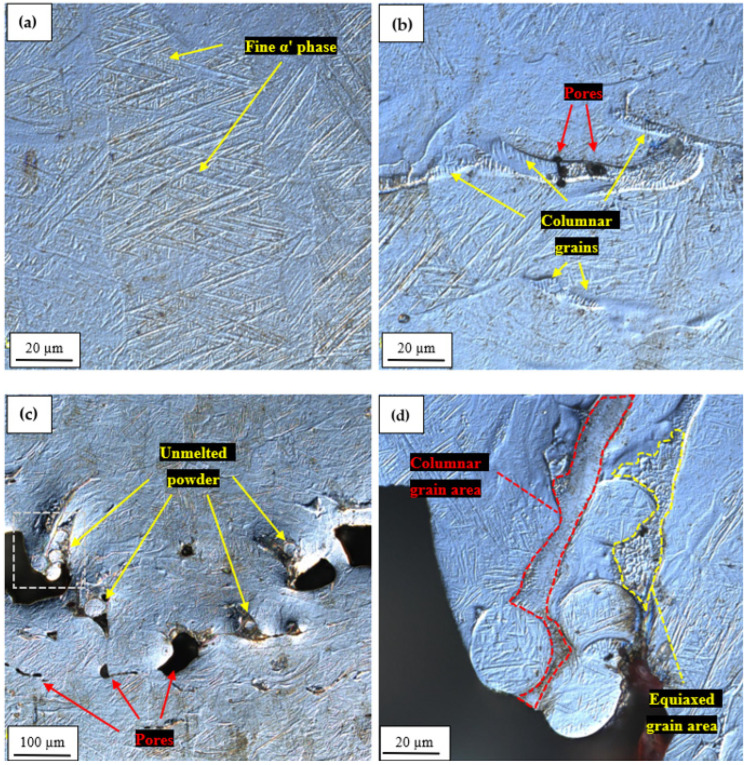
Selected microstructural images of produced samples: acicular α′ phase in sample 1 (**a**), small imperfections in sample 5 (**b**), a highly defected area in sample 6 (**c**), and higher magnification of unmelted powder particles (white box from Figure 5c) (**d**).

The microstructural images of selected samples obtained in this study have been presented in Figure 5.

The light microscope observations of the produced samples did not reveal any significant differences between them on the microstructural level. Generally, the analyzed samples are characterized by relatively low participation of imperfections, mostly in the form of porosity and singular unmelted powder particles. The exception to this are sporadic, highly porous areas, described in the further part of this section. Additionally, it has to be mentioned that no solidification cracks were reported in the investigated samples. On the microstructural level, the obtained components are characterized by the presence of ultrafine, acicular, α′ phase (Figure 5a). The formation of this phase is a direct result of the high cooling rate in the applied manufacturing process. The martensitic transformation β→α′ occurs at a cooling rate exceeding 410 K/s, and the higher the cooling rate, the finer the acicular martensite [31]. Taking into consideration that the only martensite observed in this study was ultrafine (below 1 µm width), and no presence of lath-type α′ was reported, it can be stated that the range of applied process parameters resulted in relatively low heat input [32]. Similar structures have been obtained in other studies on the selective laser melting of Ti6Al4V [33]. In some regions of sample 5, there were areas with small pores (below 5 µm) and columnar grains (Figure 5b). The columnar grains have been formed across the melted pool boundaries in the form of 5–10 µm layers, as was also reported in the case of the 3D printed Ti6Al4V alloy [32]. As it was mentioned previously, some parts of, e.g., sample 6 are characterized by the presence of highly defected areas containing large pores and numerous unmelted powder particles (Figure 5c). The pores, localized close to each other, have a size of about 80–120 µm, and in their surroundings, the unmelted powder can be observed. Below this defected area there is also a band of smaller, longitudinal pores (up to 100 µm long). Additionally, some other features have been reported in the area marked with green (Figure 5c), dashed line, which has been magnified in Figure 5d. Close to the large void, an agglomeration of unmelted, spheroidal, powder particles has been spotted. The size of the particles is within the range of 20–30 µm, and their microstructure also has an ultrafine, acicular, α′ phase pattern. In the discussed powder agglomeration, there is also a columnar grain layer (Figure 5d), similar to those previously described (Figure 5b). On the other side of the agglomeration, the small area of equiaxed grains has been identified with ultrafine grains, below 5 µm in size [34].

### 3.4. Microhardness

**Figure 6:**
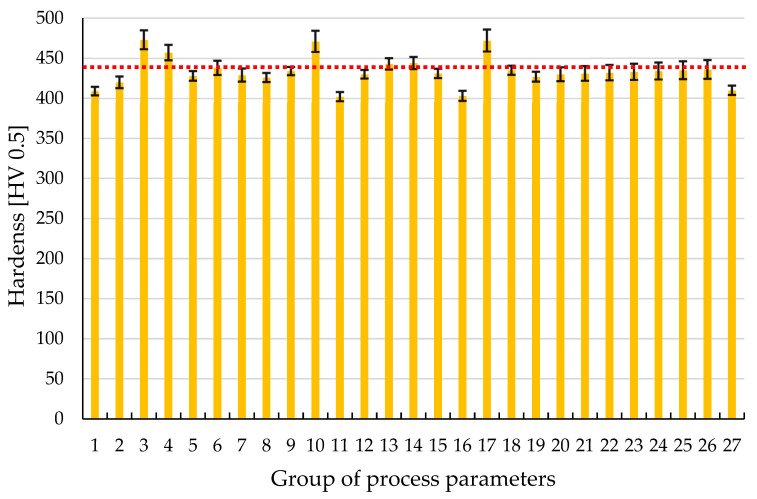
The results of measurement tests of Ti6Al4V samples produced by means of PBF-LB/M. An average value (434 HV 0.5) was marked with the use of red dased line.

The hardness measurement results are illustrated in the chart provided in Figure 6. The average hardness is 434 HV 0.5, with a standard deviation of 18 HV 0.5. Most of the results for each sample parameter fell within the specified range of deviations. This suggests that, within the investigated parameter range, there were no significant variations in the material’s hardness properties. These findings are further corroborated by the microstructural analysis. The red dashed line in the chart represents the average value across all measurements.

Data from articles on Ti6Al4V hardness studies report a hardness range between 400 and 500 HV [35,36,37,38,39]. Some cases in the literature indicate lower hardness values [40]. Variations in values may be attributed to different L-PBF process parameters that were unchanged during this study. Factors influencing hardness include exposure strategy, layer thickness, laser spot diameter, and the quality of heat dissipation in the material fusion process. Comparing the hardness of conventionally manufactured and additively manufactured materials, it is evident that the printing process allows achieving similar hardness levels to conventionally processed material after additional heat treatment. L-PBF offers precise control over material composition and microstructure, leading to enhanced mechanical properties, including comparable hardness and wear resistance to conventionally processed Ti6Al4V materials [41,42].

### 3.5. Statistical Model

Based on the 27 obtained porosity results, an equation for the quadratic response regression model was developed in accordance with the procedure outlined in Section 2. The determination of individual regression coefficients allowed for the formulation of the equation described by Relation (3), where: x_1_—power, x_2_—scanning speed, x_3_—distance between scanning vectors.

(3) $$y=-23.108+0.185x_{1}-0.026x_{2}+190.886x_{3}-0.00032x_{1}^{2}+0.000013x_{2}^{2}-750.010x_{3}^{2}\;-0.0000055x_{1}x_{2}-0.019x_{1}x_{3}-0.005x_{2}x_{3}+0.32$$

Additionally, the coefficient of determination R^2^ was calculated, which in this case is 0.45. This value indicates a poor fit between the estimated and actual values. The reason for this is the small variance in the results and the concentration of porosity values below 0.1% for most samples produced using the considered parameter groups. The values of the regression coefficients and the *p*-values are presented in Table 4.

Statistical significance (*p* < 0.05) was demonstrated by coefficients β_11_ i β_33_, which are components of the equation terms describing laser power and the distance between scanning vectors. These independent variables had the greatest impact on the estimated porosity value.

**Figure 7:**
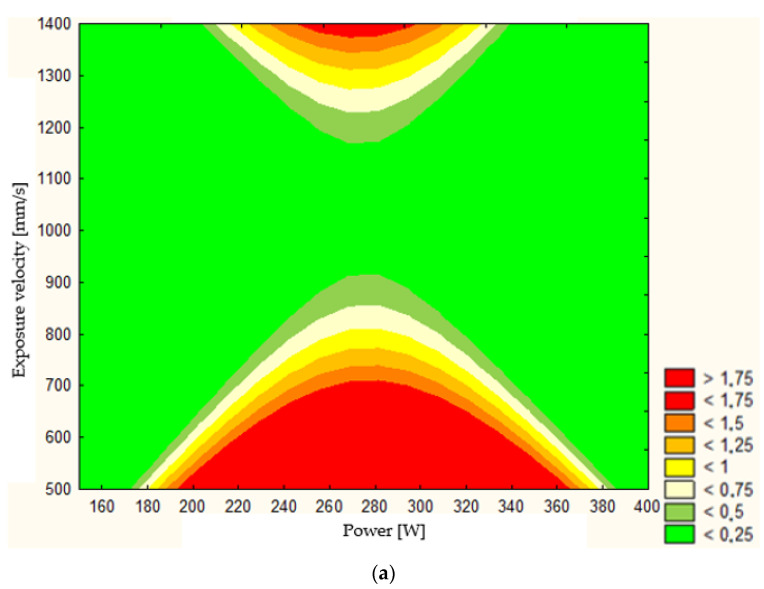

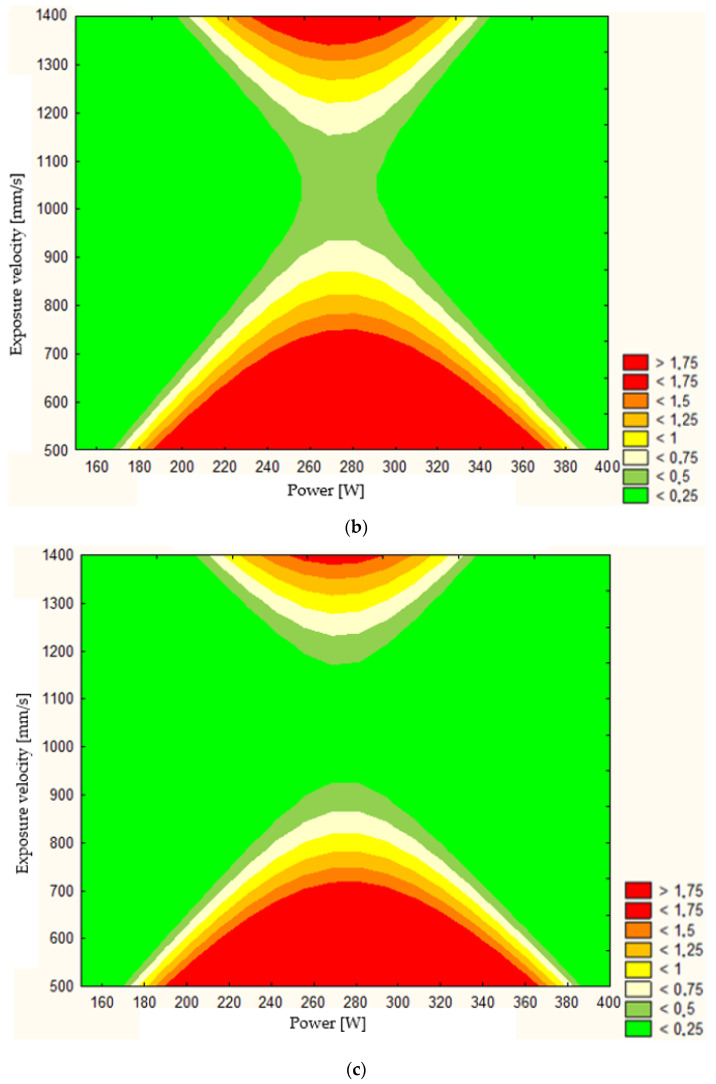
Response map for a hatch spacing of (**a**) 0.1 mm, (**b**) 0.12 mm, and (**c**) 0.14 mm.

Additionally, using Statistica software (TIBCO Software Inc., Palo Alto, CA, USA, version 13.3), it is possible to graphically represent the model’s response, assuming a constant value for one of the independent variables. Response surfaces are shown for constant values of the distance between vectors, which were considered in the studies, in Figure 7. The adopted ranges of scanning speed and laser power include the values considered in the 27 parameter groups (Table 2). On the response maps shown in Figure 7a–c, areas are highlighted where the estimated porosity values predicted by the model described in Equation (3) are indicated. Regardless of the assumed value of HHH, the shape and size of the so-called technological window—i.e., the range of parameter values within which the porosity of the produced samples will not exceed 0.5%—are defined.

The widest area of the technological window, where the estimated porosity is less than 0.25%, was observed in the maps shown in Figure 7a,c. Above 1200 mm/s and below 900 mm/s, the range of laser power values that would allow achieving favorable results in terms of low defect presence in the material structure narrows. However, it is important to note that these regions on the maps extend beyond the range of the source data. The porosity values in these regions were obtained through extrapolation, making them subject to a high degree of error. In Figure 7b, in addition to the previously described phenomena, a region was observed where a slight increase in porosity values to below 0.5% occurs within the scanning speed range of 900–1200 mm/s. The analysis of the study results provides grounds to conclude that the material under consideration exhibits low sensitivity to variations in manufacturing parameters concerning the resulting porosity. This, in turn, offers broad opportunities for shaping other properties, such as strength, during the manufacturing stage (laser powder melting) while simultaneously maintaining a low probability of structural defects in the produced components.

## 4. Conclusions

Properly adjusting the process is essential for achieving efficient, cost-effective production and ensuring high-quality microstructure and optimal mechanical properties of printed Ti6Al4V parts. A thorough comparison of the process parameters in PBF-LB/M technology for the Ti6Al4V titanium alloy facilitated the development of a mathematical model aimed at optimizing parameter selection. Based on the results obtained, the following conclusions were drawn:

1. The material porosity for different additive manufacturing process parameters ranges from 0.005% to 1.54%. The highest porosity was obtained using the 11th parameter group (P = 275 W, v = 1210 mm/s, H = 0.12 mm). For these parameters, the energy density per unit volume of material is 63.13 J/mm^3^.
2. The lowest porosity was achieved with the 7th parameter group. The parameter values used in this group were P = 302.5 W, v = 990 mm/s, and H = 0.14 mm. The calculated volumetric energy density is 72.75 J/mm^3^.
3. The analysis indicates that there are only minor differences in energy density between the highest and lowest porosity values. This suggests that the relationship between process parameters and porosity is not strongly influenced by variations in energy density alone. Instead, other factors, such as the interplay between laser power, exposure velocity, and hatching distance, may play a more significant role in determining porosity.
4. Using selected images, the material’s microstructure was presented. In most cases, the material’s microstructure is similar, and material defects are caused by gas porosity or lack of fusion of individual grains.
5. Hardness measurements of the material showed an average value of 434 ± 18 HV0.5. The material’s hardness does not significantly change with varying additive manufacturing process parameters.
6. Using a mathematical model, response maps were generated for selecting additive manufacturing parameters using the SLM technique. Analyzing these maps reveals that a range of parameters ensuring low porosity (beneficial for the material’s strength properties) can be achieved with various combinations of parameters.

## Figures and Tables

**Table 1:** Chemical composition of Ti6Al4V powder.

Element	Ti	Al	V	Fe	O	C	N	H
Weight (%)	Bal.	5.5–6.75	3.5–4.5	<0.3	<0.2	<0.1	<0.05	<0.015

**Table 2:** Combination of printing parameters.

Sample	Power [W]	Layer Thickness [mm]	Exposure Velocity [mm/s]	Hatching Distance [mm]	Energy Density [J/mm^3^]
1	302.5	0.03	1210	0.14	59.52
2	275	0.03	1210	0.14	54.11
3	247.5	0.03	1210	0.14	48.70
4	302.5	0.03	1100	0.14	65.48
5	275	0.03	1100	0.14	59.52
6	247.5	0.03	1100	0.14	53.57
7	302.5	0.03	990	0.14	72.75
8	275	0.03	990	0.14	66.14
9	247.5	0.03	990	0.14	59.52
10	302.5	0.03	1210	0.12	69.44
11	275	0.03	1210	0.12	63.13
12	247.5	0.03	1210	0.12	56.82
13	302.5	0.03	1100	0.12	76.39
14	275	0.03	1100	0.12	69.44
15	247.5	0.03	1100	0.12	62.50
16	302.5	0.03	990	0.12	84.88
17	275	0.03	990	0.12	77.16
18	247.5	0.03	990	0.12	69.44
19	302.5	0.03	1210	0.1	83.33
20	275	0.03	1210	0.1	75.76
21	247.5	0.03	1210	0.1	68.18
22	302.5	0.03	1100	0.1	91.67
23	275	0.03	1100	0.1	83.33
24	247.5	0.03	1100	0.1	75.00
25	302.5	0.03	990	0.1	101.85
26	275	0.03	990	0.1	92.59
27	247.5	0.03	990	0.1	83.33

**Table 3:** Printing parameters series—7 and 11.

Sample	Power [W]	Layer Thickness [mm]	Exposure Velocity [mm/s]	Hatching Distance [mm]	Energy Density [J/mm^3^]	Porosity [%]
7	302.5	0.03	990	0.14	72.75	0.005
11	275	0.03	1210	0.12	63.13	1.54

**Table 4:** The values of the regression coefficients and their statistical significance levels.

Coefficient	Value	*p* Value
β_0_	−23.108	0.275574
β_1_	0.185	0.055769
β_11_	−0.00032	0.043106
β_2_	−0.026	0.277420
β_22_	0.000013	0.174263
β_3_	190.886	0.055563
β_33_	−750.010	0.014936
β_12_	−0.0000055	0.859799
β_13_	−0.019	0.912609
β_23_	−0.005	0.902713

## Data Availability

The research data are available upon request (janusz.kluczynski@wat.edu.pl).
